# Measuring Coverage in MNCH: Challenges in Monitoring the Proportion of Young Children with Pneumonia Who Receive Antibiotic Treatment

**DOI:** 10.1371/journal.pmed.1001421

**Published:** 2013-05-07

**Authors:** Harry Campbell, Shams el Arifeen, Tabish Hazir, James O'Kelly, Jennifer Bryce, Igor Rudan, Shamim Ahmad Qazi

**Affiliations:** 1 Centre for Population Health Sciences, University of Edinburgh, Edinburgh, United Kingdom; 2 International Centre for Diarrhoeal Disease Research, Bangladesh, Dhaka, Bangladesh; 3 Children's Hospital, Pakistan Institute of Medical Sciences, Islamabad, Pakistan; 4 Department of International Health, Johns Hopkins Bloomberg School of Public Health, Johns Hopkins University, Baltimore, Maryland, United States of America; 5 Department of Maternal, Newborn, Child and Adolescent Health, World Health Organization, Geneva, Switzerland; Wellcome Trust Senior Research Fellow in Clinical Science, UCL Reader in International Child Health, Honorary Consultant, Great Ormond Street Hospital for Children, United Kingdom

## Abstract

Pneumonia remains a major cause of child death globally, and improving antibiotic treatment rates is a key control strategy. Progress in improving the global coverage of antibiotic treatment is monitored through large household surveys such as the Demographic and Health Surveys (DHS) and the Multiple Indicator Cluster Surveys (MICS), which estimate antibiotic treatment rates of pneumonia based on two-week recall of pneumonia by caregivers. However, these survey tools identify children with reported symptoms of pneumonia, and because the prevalence of pneumonia over a two-week period in community settings is low, the majority of these children do not have true pneumonia and so do not provide an accurate denominator of pneumonia cases for monitoring antibiotic treatment rates. In this review, we show that the performance of survey tools could be improved by increasing the survey recall period or by improving either overall discriminative power or specificity. However, even at a test specificity of 95% (and a test sensitivity of 80%), the proportion of children with reported symptoms of pneumonia who truly have pneumonia is only 22% (the positive predictive value of the survey tool). Thus, although DHS and MICS survey data on rates of care seeking for children with reported symptoms of pneumonia and other childhood illnesses remain valid and important, DHS and MICS data are not able to give valid estimates of antibiotic treatment rates in children with pneumonia.

*This paper is part of the* PLOS Medicine *“Measuring Coverage in MNCH” Collection.*

## Introduction

Pneumonia has been the largest single cause of child death over the 2000–2015 Millennium Development Goal period [1],[2], and despite large falls in global under-five mortality, pneumonia remains the major single cause of child death in the post-neonatal period [3]. Recent estimates suggest that pneumonia accounted for about 0.32 million deaths in the first month of life and 1.1 million post-neonatal child deaths in 2010—over 14% of all child deaths under five years of age. These estimates, together with an analysis of the rate of fall of cause-specific child mortality, suggest that efforts to reduce child deaths from pneumonia will have to be accelerated if the target of Millennium Development Goal 4—to reduce child mortality by two-thirds between 1990 and 2015—is to be met [3]. Moreover, pneumonia accounts for a substantial percentage of all paediatric out-patient attendances, in-patient admissions, and antibiotic prescriptions in health services in low- and middle-income countries (bacterial pneumonia is the major cause of severe episodes of pneumonia and death from pneumonia) and so places a large burden on these health services and on the families involved [4].

Several effective interventions are available to tackle the challenge of childhood pneumonia, as recently summarised in a series of review articles commissioned as part of the Global Action Plan for the Prevention and Control of Pneumonia [4],[5]. Key amongst these interventions are immunisation and correct case management of young children with pneumonia who present to trained health workers. Both of these strategies have been shown to be effective through controlled trials [6],[7]. However, although there are robust and accurate mechanisms to measure the performance and progress of immunisation programmes, there are no well-established monitoring methods for (community) case management programmes.

The two major interventions that reduce mortality from bacterial pneumonia are antibiotic treatment (for all cases) and oxygen therapy (in those children who have hypoxaemia). Thus, programme monitoring of case management programmes at (sub-) national, regional, and global levels requires the accurate measurement of the percentage of children with pneumonia in a defined population who receive antibiotic therapy.

Unfortunately, caregivers of sick children with pneumonia often do not seek antibiotic treatment from trained health providers. Findings from eight studies from seven low- and middle-income countries that interviewed caregivers of young children with pneumonia suggest that caregivers sought care from an appropriate health provider who could give correct antibiotic treatment in only about half of pneumonia episodes [8]–[15]. These findings highlight the need for community-based rather than hospital-based studies to measure antibiotic treatment rates in children with pneumonia if a true population-based estimate is to be made (rather than a biased estimate based on the unrepresentative subgroup of children whose caregivers seek care). Furthermore, the availability of first and second line antibiotics within first level health facilities in low- and middle-income countries may be poor and may vary markedly across institutions, again suggesting that a population-based survey is required.

Programme monitoring therefore needs to identify a representative group of children who have recently had an episode of pneumonia and then investigate what proportion of these children received (correct) antibiotic treatment. In recent years, the Demographic and Health Surveys (DHS) and the United Nations Children's Fund Multiple Indicator Cluster Surveys (MICS) have undertaken these processes in low- and middle-income countries. Specifically, these surveys ask caregivers to recall symptoms and signs of pneumonia in their children, which provides the denominator of pneumonia cases, and collect data from caregivers on antibiotic treatments received, which allows the antibiotic treatment rate to be estimated. In this review, which is part of the *PLOS Medicine* “Measuring Coverage in MNCH” Collection, we consider how our current understanding of the epidemiology of childhood pneumonia can inform the design and interpretation of surveys seeking to monitor antibiotic treatment rates.

## Disease Prevalence and Survey Sample Size

It is reasonable to assume that the data used to monitor antibiotic treatment rates will not be widely available from cohort studies but will be measured in cross-sectional surveys. The power of such surveys can be roughly estimated by assuming that there is no seasonality of pneumonia incidence and that the duration of pneumonia symptoms is one week on average. A cross-sectional survey of 1,000 young children that enquires about symptoms of pneumonia that were present in the past two weeks should identify approximately 18 caregivers whose child truly had an episode of pneumonia at some point within this recall period. This two-week period prevalence is calculated from a summary estimate of pneumonia incidence of 300 cases per 1,000 children per year in low- and middle-income countries based on a systematic review of population-based cohort studies using standard case definitions that are consistent with the World Health Organization Integrated Management of Childhood Illness case definition of pneumonia [16]. This summary estimate equates to about six new episodes per week per 1,000 children. Children with new episodes that arise over a three-week period will have pneumonia symptoms falling within the two-week period if the child is symptomatic for one week.

Efforts can then be made to establish whether the children with pneumonia received appropriate antibiotic treatment. To estimate a treatment rate of 50% with a precision of ±5% (95% confidence interval of 45%–55%) would require 385 children with pneumonia to be surveyed, and this in turn would require a survey sample size of about 20,000 children. Thus, surveys of many thousand caregivers of young children are required to generate a sufficiently large denominator of “children with pneumonia” from which to measure antibiotic treatment rate. The only such surveys that are conducted widely in low- and middle-income countries at this scale are the DHS and MICS surveys. Both types of survey collect information on children with cough accompanied by rapid or difficult breathing that is due to a problem in the chest. DHS refers to these children as having “symptoms of acute respiratory infection”; MICS refers to them as “suspected” cases of pneumonia. Neither of these survey programmes claims that actual cases of pneumonia are measured through the questions included in the surveys.

## How Well Do Reported Symptoms and Signs of Pneumonia Indicate the Presence of True Pneumonia?

We have previously reported that in conditions of low disease prevalence it is very challenging to obtain an accurate estimate of the true number of episodes of a condition from a screening test—in this case eliciting caregiver report of symptoms of pneumonia or “suspected pneumonia” based on clinical signs recognised by the caregiver (as in DHS and MICS surveys) [17]. For simplicity, throughout the rest of this paper, we will refer to these as cases of “suspected pneumonia”.

Table 1 shows a schematic distribution of cases of “true pneumonia” (true disease) according to caregiver report of “suspected pneumonia” (reported symptoms) and true disease status. The sensitivity, specificity, positive and negative predictive values of the survey (test characteristics), and disease prevalence can all be calculated from the numbers of children in cells *a–d* of this table (see Box 1). As we will now discuss, plausible values of pneumonia prevalence and of sensitivity and specificity can be inserted into these 2×2 tables, and the findings can be used to better understand the output of DHS and MICS surveys.

**Table 1:** Distribution of cases of “true pneumonia” (true disease) according to caregiver report of “suspected pneumonia” (reported symptoms) and true disease status.

Reported Symptoms	True Disease		
	Present	Absent	Total
Present	*a*	*b*	
Absent	*c*	*d*	
Total			

Cell *a* represents children with “true pneumonia” (true positives) whose caregiver gave a report of “suspected pneumonia” (test positive). Cell *b* represents children without pneumonia (true negatives) whose caregiver gave a report of “suspected pneumonia” (test positive). Cell *c* represents children with “true pneumonia” (true positives) whose caregiver did not give a report of “suspected pneumonia” (test negative). Cell *d* represents children without pneumonia (true negatives) whose caregiver did not give a report of “suspected pneumonia” (test negative).

**Box 1. Calculation of Test Characteristics**

If we consider the caregiver report of reported symptoms and signs of pneumonia (referred to below as “suspected pneumonia”) as defined by DHS and MICS survey guidelines to be a test of true pneumonia status in the child, then:

- The sensitivity of the caregiver report of “suspected pneumonia” (test) is given by *a*/(*a*+*c*);
- The specificity of the caregiver report of “suspected pneumonia” (test) is given by *d*/(*b*+*d*);
- The positive predictive value of the caregiver report of “suspected pneumonia” (test) is given *a*/(*a*+*b*);
- The negative predictive value of the caregiver report of “suspected pneumonia” (test) is given by *d*/(*c*+*d*); and
- The disease prevalence is given by (*a*+*c*)/(*a*+*b*+*c*+*d*).

Our first example (Table 2) considers a scenario where 1,000 caregivers are surveyed, the test sensitivity is 80%, and the test specificity is 85%. This corresponds to roughly the level of sensitivity that has been reported for a trained health worker assessment of pneumonia at a health centre based on clinical signs but assumes a higher level of specificity than is usually achieved in these circumstances. It is unlikely that the performance of caregiver report of “suspected pneumonia” will reach these levels of sensitivity and specificity, and this scenario is therefore likely to give an over-optimistic picture of the ability of surveys based on caregiver report to discriminate true pneumonia. Table 2 illustrates that in this scenario there would be 161 reports of “suspected pneumonia” and 18 cases of true pneumonia among the children of the 1,000 caregivers surveyed—a ratio of reported “suspected pneumonia” to true pneumonia of 8.9∶1 (161/18). Thus, “suspected pneumonia” in this setting is a very inaccurate measure of true pneumonia frequency. Furthermore, of the 161 cases of “suspected pneumonia” for which the caregiver would be questioned about receipt of antibiotic treatment, only 14/161 (8.7%, the positive predictive value of the survey tool) would have true pneumonia, making “suspected pneumonia” a very unreliable denominator from which to calculate antibiotic treatment rate. If all 14 children with true pneumonia correctly received antibiotic treatment and all the cases without true pneumonia correctly did not receive antibiotic treatment, then this would be recorded as an antibiotic treatment rate of about 9%, and programme efforts to substantially increase this rate would only serve to promote the over-prescription of antibiotics.

**Table 2:** Distribution of cases of “true pneumonia” according to caregiver report of “suspected pneumonia” (test) and true disease status when test sensitivity is 80% and specificity is 85%.

Reported Symptoms	True Disease		
	Present	Absent	Total
Present	14	147	161
Absent	4	835	839
Total	18	982	1,000

It is possible that, due to a different “case mix” (a different proportion of completely well children) at the household and at the health centre level, the specificities of “suspected pneumonia” in household surveys could be higher than those based on clinical signs recorded by trained health workers at the health centre. Thus, in Tables 3 and 4, we illustrate examples with higher test specificities. As the test specificity rises to very high levels (95% and 99%, respectively), the ratio of reported “suspected pneumonia” to true pneumonia falls to 3.5∶1 (63/18) and 1.3∶1 (24/18), respectively—still overestimates of pneumonia prevalence but much less inaccurate. In addition, as the specificity increases, the proportion of “suspected pneumonia” that is true pneumonia (the positive predictive value of the tool) rises to 22% (14/63) and 58% (14/24), respectively. Thus, maximising test specificity has the potential to make large improvements to the validity of the denominator that is used to measure pneumonia treatment rates. An increase in specificity from 80% to 99% increases the proportion of “suspected pneumonia” that is truly pneumonia (the positive predictive value) from less than 9% (Table 2) to approximately 58% (Table 4).

**Table 3:** Distribution of cases of “true pneumonia” according to caregiver report of “suspected pneumonia” (test) and true disease status when test sensitivity is 80% and specificity is 95%.

Reported Symptoms	True Disease		
	Present	Absent	Total
Present	14	49	63
Absent	4	933	937
Total	18	982	1,000

**Table 4:** Distribution of cases of “true pneumonia” according to caregiver report of “suspected pneumonia” (test) and true disease status when test sensitivity is 80% and specificity is 99%.

Reported Symptoms	True Disease		
	Present	Absent	Total
Present	14	10	24
Absent	4	972	976
Total	18	982	1,000

By contrast, decreasing test sensitivity from 80% to 60% with a fixed specificity of 95% has only a modest impact on the ratio of reported “suspected pneumonia” to true pneumonia, which falls only slightly from 3.5∶1 (63/18) (Table 3) to 3.3∶1 (60/18) (Table 5). Moreover, the positive predictive value falls only slightly from 22% (4/63) (Table 3) to 18% (11/60) (Table 5). Since increasing test specificity is usually linked to falling test sensitivity, it is clear that maximising test specificity should be prioritised in the test design. We will briefly discuss how test specificity might be maximised at the end of this review.

**Table 5:** Distribution of cases of “true pneumonia” according to caregiver report of “suspected pneumonia” (test) and true disease status when test sensitivity is 60% and specificity is 95%.

Reported Symptoms	True Disease		
	Present	Absent	Total
Present	11	49	60
Absent	7	933	940
Total	18	982	1,000

There are actually very few published reports of the sensitivity and specificity of caregiver reports of symptoms and signs of pneumonia for the discrimination of true pneumonia. However, using the mean estimate of sensitivity (31%) and specificity (91%) of caregiver report of fast or difficult breathing for prediction of true pneumonia (diagnosed by a study physician) from two community-based studies in Gambia [18] and Pakistan [19] would yield 94 reported cases of “suspected pneumonia” among 1,000 children. This represents a ratio of “suspected pneumonia” to true pneumonia of 5.2∶1 (94/18) and a positive predictive value of only 6.4% (6/94). As discussed elsewhere in this Collection, studies undertaken in Pakistan and Bangladesh provide additional data from both hospital-based and community-based studies to further assess the important issue of detection of true pneumonia [20].

Importantly, as noted earlier, the data on the sensitivity and specificity of pneumonia reporting that is needed to determine the prevalence of true pneumonia should ideally come from community-based studies because hospital-based clinic studies may overestimate sensitivity and underestimate specificity due to the different case mix at the hospital level (pneumonia cases tend to be more severe on average in hospitals than in the community, and non-pneumonia cases tend to be less healthy and more likely to have other causes of difficulty breathing). Moreover, because the discriminative power of caregiver reports depends largely on caregiver recognition of symptoms and signs of pneumonia, it is also likely to be influenced greatly by contextual factors such as the level of maternal education and prior exposure to relevant health education messages. Thus, the interpretation of trends in antibiotic treatment data over time will be complicated in settings where there have been temporal trends in education levels or health education interventions or where serial surveys have been conducted in different populations.

## Another Strategy for Improving the Positive Predictive Value of Surveys

In addition to increasing test specificity, another strategy that should improve the proportion of “suspected pneumonia” reports that truly represent cases of pneumonia (positive predictive value) is to increase the disease prevalence detected in DHS and MICS surveys. This could be achieved by conducting surveys during the peak pneumonia season. Alternatively, if the recall period were to be increased from two weeks to four weeks (or eight weeks), then 30 (or 54) reports of pneumonia symptoms and signs would be expected, rather than 18 reports. Tables 6 and 7 show that with the same levels of sensitivity (80%) and specificity (95%) as in the example in Table 3, these longer recall periods would yield 72 (or 90) reported cases of “suspected pneumonia” and 30 (or 54) cases of true pneumonia among 1,000 children. Thus, the ratio of “suspected pneumonia” to true pneumonia would be about 3∶1 (or 1.7∶1) (compared to 3.5∶1 in Table 3), and the proportion of “suspected pneumonia” that is true pneumonia would be 33% (or 48%) rather than 22% as in Table 3.

**Table 6:** Distribution of cases of “true pneumonia” according to caregiver report of “suspected pneumonia” (test) and true disease status when test sensitivity is 80% and specificity is 95% with a four-week recall period.

Reported Symptoms	True Disease		
	Present	Absent	Total
Present	24	48	72
Absent	6	922	928
Total	30	970	1,000

**Table 7:** Distribution of cases of “true pneumonia” according to caregiver report of “suspected pneumonia” (test) and true disease status when test sensitivity is 80% and specificity is 95% with an eight-week recall period.

Reported Symptoms	True Disease		
	Present	Absent	Total
Present	43	47	90
Absent	11	899	910
Total	54	946	1,000

If we use the data from Gambia [18] and Pakistan [19] on test specificity and sensitivity combined with longer recall periods, the proportion of “suspected pneumonia that is true pneumonia (positive predictive value) would rise from 6.4% to 9.8% (or 16.7%) based on four-week (or eight-week) recall, respectively. Because these predictions are based on the assumption that test performance would not change with the longer recall period, it will be important to test this assumption (see [20]) before any such increase in recall period is introduced into surveys.

## A Combined Strategy to Improve the Identification of True Pneumonia Episodes from Maternal Report of “Suspected Pneumonia”

Finally, we can consider the effect of combining an improved test specificity and an increased recall period. Using the published Gambia [18] and Pakistan [19] data, if study recall could be increased from two to four or eight weeks and test specificity increased from 91% to 97%, then the proportion of “suspected pneumonia” that is true pneumonia (the positive predictive value) would rise by almost an order of magnitude from 6.4% to 23.7% or 56.9% for four- or eight-week recall, respectively. The field studies in Pakistan and Bangladesh [20] provide further data from which to estimate the improvements that could be expected from attempts to improve survey instruments.

## Are DHS and MICS Surveys Suitable Tools for Monitoring Antibiotic Treatment of Childhood Pneumonia?

A research priority setting exercise that involved a large number of doctors from low- and middle-income countries recently listed improving the community case management of pneumonia and identifying barriers to and improving access to antibiotic treatment as among the top research priorities likely to contribute to achievement of Millennium Development Goal 4 [21]. The identification of these research priorities reflects the need to reduce childhood pneumonia if childhood mortality is going to be reduced, and supports the self-evident need to develop a robust programme indicator—the antibiotic treatment rate—to monitor local, national, and global progress in increasing the coverage of this essential pneumonia intervention. Measurement of this indicator has to be community-based (to capture the many pneumonia cases that do not attend health services for treatment) and large (to include enough pneumonia cases to give precise estimates). DHS and MICS surveys are the only tools that fit these requirements and that are conducted widely in developing countries. It is therefore important to assess their suitability for this purpose, since estimates of pneumonia prevalence and antibiotic treatment coverage based on these surveys will be influential in guiding national and international decisions about programmes to control pneumonia deaths.

### The Validity of DHS and MICS “Suspected Pneumonia” Survey Data for the Estimation of the Prevalence of True Pneumonia

In circumstances of low pneumonia prevalence (such as found with the two-week recall of pneumonia episodes included in current surveys), even when the sensitivity and specificity of “suspected pneumonia” as a test for true pneumonia is very high (>90%), the estimates of the number of pneumonia episodes based on DHS and MICS survey data will be greatly inflated, and most reported episodes will be false positives [17]. DHS and MICS survey questions were not designed to estimate pneumonia prevalence, and current DHS and MICS guidelines advise against the use of data in this way. Our review reinforces these recommendations.

### The Validity of DHS and MICS “Suspected Pneumonia” Survey Data for the Estimation of the Antibiotic Treatment Rate for Pneumonia

The findings we present in this review (Tables 1–7) have important implications for the use of existing DHS and MICS survey data in monitoring antibiotic treatment rates. The DHS/MICS antibiotic treatment indicator for “suspected pneumonia” is often used as a proxy indicator for a pneumonia treatment indicator. For this indicator to give accurate information that is useful for programme planning, we need to have a denominator as close to true pneumonia as possible. A denominator of “suspected pneumonia” in which most cases are not true pneumonia makes interpretation of this indicator problematic, and action based on adoption of this indicator alone could drive over-prescription of antibiotics.

It is clear from the examples we work through in this review and from underlying theory based on known epidemiology of pneumonia [17] that the discriminative power of survey instruments needs to be improved. This discriminative power is constrained by the inherent limited ability of caregivers to correctly recognise and report symptoms and signs of pneumonia. Within this constraint, it is possible to increase test specificity (at the expense of test sensitivity) by adding a few additional symptoms or signs to survey questionnaires that show good predictive power or by employing a “pneumonia score”, as reported elsewhere in this Collection [20]. In this latter approach, a survey would include questions about a series of signs or symptoms of pneumonia, and one mark would be awarded for each sign or symptom that is reported. A threshold score level defined by a favourable combination of sensitivity and specificity levels could then be selected for use. However, it is possible that the underlying discriminative power of this approach will remain constrained since it relies on caregiver recognition. One way to overcome this constraint and to improve overall discriminative power might be to adopt a new approach for questioning caregivers that relies on video recognition [20]. Such an approach would operate via visual recognition memory rather than via auditory recognition memory, which may promote better recognition and recall [22]. This argument could also be applied to the measurement of the antibiotic treatment rate indicator. Thus, “pill boards” or digital formats could be shown to caregivers that illustrate a range of local drugs, to promote correct recall of antibiotic prescriptions.

It could be argued that although the interpretation of the absolute value of the antibiotic treatment rate indicator is very problematic, there may still be considerable utility in using its relative value to track trends over time (and to compare across countries) for programme planning purposes. However, these data should be interpreted with caution, since contextual factors are likely to influence results significantly, and these may vary by time and place independent of trends in antibiotic treatment rates.

### The Validity of DHS and MICS “Suspected Pneumonia” Survey Data for the Estimation of Appropriate Care Seeking for Pneumonia

The care-seeking indicator for “suspected pneumonia” that is found in DHS/MICS surveys remains valid. “Suspected pneumonia” based on simple signs that caretakers can understand and that programmes can use is an appropriate denominator for this indicator, as the aim is to encourage all these children to be assessed by a health provider whether or not they actually have pneumonia.

## Future Research and Prospects

Given the importance of antibiotic treatment rates as a programme indicator, there is an urgent need for more research to measure the sensitivity and specificity of “suspected pneumonia” as defined in DHS and MICS surveys for the identification of true pneumonia episodes. The sensitivity and specificity of “suspected pneumonia” needs to be measured in a range of settings and with questions based on a range of recall periods from two weeks to several months, and must also be compared to the performance of new approaches such as those described above. Hazir et al. provide some first estimates of these parameters based on two- and four-week recall and estimate the impact on test performance of some new survey methods [20]. Consideration should also be given to the feasibility of including in future surveys some assessment of whether the prescribed antibiotic was actually taken correctly by the child.

In addition to optimising existing means of determining antibiotic treatment rates for pneumonia, recent developments in eHealth and mHealth (health care supported by electronic processes and communication and by mobile devices, respectively) applications in low- and middle-income countries and their use, for example, in surveillance of influenza episodes [23] may mean that novel real-time measurement of child health programme indicators will soon be feasible in some settings. Finally, in the short term, digital illustrations of local treatments or of children with signs of pneumonia that are recognised by local caregivers should be technically feasible and could facilitate accurate data capture, storage, and transmission for analysis [24], thereby helping to improve the way we monitor antibiotic treatment coverage among young children with pneumonia.

**Key Points**

- Large household surveys are required to identify recent cases of pneumonia as a denominator from which antibiotic treatment rates for pneumonia can be estimated.
- At the low levels of pneumonia prevalence found in household surveys, most of the children identified with “suspected pneumonia” will not have true pneumonia, even when survey tools with very high sensitivity and specificity are used.
- This inflation of the denominator of the antibiotic treatment rate will make the treatment rate appear falsely low and could lead to incorrect programme decision making.
- In theory, the performance of DHS/MICS survey tools can be improved by increasing test specificity and/or by increasing pneumonia period prevalence (by increasing the recall period or by conducting the survey in the peak pneumonia season), but this prediction needs testing.
- Alternate approaches to measuring the antibiotic treatment rate should also be considered, including those that make use of digital formats to facilitate pneumonia recognition and recall of antibiotic treatment by caregivers.

## Abbreviations

DHS: Demographic and Health Surveys
MICS: Multiple Indicator Cluster Surveys
